# The Role of Microbes and Their Chemical Products in the Causation of Disease

**Published:** 1886-10

**Authors:** James Law

**Affiliations:** Professor of Veterinary Science, Cornell University, N. Y.


### ART. XVIII.—THE ROLE OF MICROBES AND THEIR CHEMICAL PRODUCTS IN THE CAUSATION OF DISEASE.

BY JAMES LAW, F. R. C. V. S.

*Professor of Veterinary Science, Cornell University, N. Y.*

In the present article it is not intended to demonstrate that the existence and propagation of contagious disease are due to the presence and transference of microbes or organized particles of living matter known as germs. This much may be freely accepted as absolutely demonstrated for a certain number of infectious maladies, while it is in the highest degree probable for the remainder. In the case of anthrax, chicken cholera, tuberculosis and glanders, the artificial culture of the bacterium through eight, ten or twenty successive flasks, each in succession charged with as much infecting material only as will adhere to the extreme tip of a small needle, without any appreciable loss of potency in the pathogenic quality, demonstrates a self-propagating power in a given line, which is indicative of continuous generation and life, rather than of a simple chemical change occurring in dead matter.

The Liebig theory of continuous chemical change under a given influence, as illustrated by the effect of incandescence on inflammable bodies, or of diastase on complex matter like starch, is not so acceptable as that of simple self-generation and propagation. The inflammable gas or explosive gunpowder may remain for an indefinite time in contact with the oxygen of the air without becoming incandescent. It is only when the requisite degree of heat has been generated in contact with the two, that their destructive combination is effected. With a given accession of heat this action is invariable, and once started the combustion combination goes on uninterruptedly until the whole of the inflammable material has been consumed. But it is far different with the disease virus. The temperature of a mammal is not alone sufficient to start it into action, nor is it always necessary. The virus multiplies out of the body at ordinary temperatures in different cases. The virus which is deadly to the bovine races is entirely harmless to the ovine or equine, though all alike have the same or a closely allied temperature, all take in similar food, all produce liquids and tissues closely allied in chemical composition, and capable in many cases, when once removed from the living body, of being acted on by the virus to which the animal supplying such tissue was insusceptible, and of increasing such virus indefinitely. These and other equally valid objections arise toward the diastase illustration: The conditions of life of the susceptible and insusceptible animal of a given species are the same; food, drink, ingesta, digestion, assimilation, disassimilation, secretion and excretion are the same in both so far as chemical investigation can ascertain, yet the behavior of the two in the presence of a specific virus is as different as light and darkness. A mere chemical product inducing change in another chemical material would act upon that in a corresponding manner wherever they might be brought in contact. The theory that the inability of the virus to establish its action in one case, is due to the previous abstraction from the system of some compound necessary to its action is negatived by the fact that the infusion of the tissues of that same animal body, freely sustains and increases the virulent matter. The doctrine that the exhibition is due to the deposition in the body of an insusceptible animal of some product antagonistic to such chemical action, is disproved by the same fact, as also by the further consideration that such new and antagonistic product would be inoperative if laid up in an insoluble form, and would be speedily carried out of the system by the emunctories if soluble; also that the chemical action of the virus on such preservation product must gradually use it up so that the system would once more be laid open to the attacks of the disease, whereas, as a rule, the longer the exposure of the animal system to a given disease-poison, the more perfect is its power of resistance and self-protection.

It may be assumed then that in dealing with the contagious disease, we are dealing with a germ or seed which propagates itself, like any other seed, when furnished with appropriate food under suitable conditions of life, and which becomes injurious largely in proportion to its abundance. Having reached this position, we are justified in ascribing to these living germs the essential characteristics of all living organisms.

*First—There is no new creation of disease germs.*—The doctrine of spontaneous generation may now be said to be definitely exploded. In every instance where organic matter has been absolutely deprived of life, by heat or otherwise, and carefully guarded against the introduction of new germs it has remained absolutely lifeless and unchanged. What occurs in the cotton-plugged sterile flask is equally true of the world at large. The fermenting wort and the rising dough convey to the mind at once the previous introduction of yeast; the waving grainfield is demonstration of the previous sowing of seed; the sight of a herd of calves carries the mind inevitably back to their sires and dams. To see a germ operating in the production of a disease therefore is to receive the assurance that that germ has had parent germs, and that when these parent germs were found under the same conditions of life, they too had produced that disease in time past. It follows that no contagious disease produced by a living germ can have arisen spontaneously and developed that germ *de novo*, and thus we are fully justified in the presence of a single case of contagious disease in making enquiry as to antecedent cases from which this has been more or less directly derived.

This does not invalidate the fact that a germ may throughout one or more generations lose much of its pathogenic potency, as in Pasteur's cultivated bacteria, or in seasons when a grave disease shows for time an unwonted originancy, yet in all such cases the direct line of generative succession is unbroken and the final cases are as surely derived from the antecedent one as if the intervening cases had been violent and fatal. A disease germ is therefore descended from other germs, and a contagious disease, from a pre-existing contagious disease.

*Second—The Disease-Germ must feed.*—A living organized being can only live, grow, and multiply through the appropriation of food from surrounding material. The disease-germ therefore living amongst the animal fluids must assimilate portions of these fluids to maintain its existence and propagate its kind. In this way it may impair health by abstracting from the blood and lymph materials necessary to the maintenance of the normal functions of its host; or by entering the blood-globules or tissue-nuclei and abstracting from their proper substance, elements that are essential to their integrity. The rapid appropriation of the oxygen of the animal fluids by its bacillus anthracis, has been held to be an important factor in causing the early and high mortality of anthrax, and one can easily conceive of irreparable injury through this devouring appetite of the disease-germ.

*Third—The Disease-Germ must excrete.*—The necessary sequence of feeding is excretion; of assimilation is disassimilation. This is no less a necessity in the lowest forms of life, than to the highest. Thus the beer yeast consumes the sugar in the malt and, after using it for its own nourishment throws out with the liquid carbonic acid and alcohol. Long since Panum drew attention to the fact that albuminous matters in a state of putrefaction developed a soluble poisonous body. Fazzie and a number of others followed in the same track and finally Selim demonstrated that a number of such poisonous *ptomaines*, were veritable organic alkaloids. Now to day we know that all such putrefactions are caused by bacteria and vibriones and that the *ptomaines* are the products of the vital processes of these minute organisms.

While the *ptomaines* of certain bacteria are deadly poisons, those of others are comparatively harmless, and hence many fungoid and bacterial organisms growing on or in the animal body are not incompatible with good health.

It follows that bacteria &c., may cause disease through the deleterious action of these secretions or excretions as well as through their abstraction from the animal fluids of materials essential to life. Practically this was demonstrated by Toussaint and Paul-Bert, who respectively filtered the virulent liquids and sterilized them by compressed oxygen, and found that the devitalized liquids administered, in large doses proved more rapidly fatal than did a moderate dose of the original virus. Moreover the sterilized liquid began its poisonous action at once, and the disease-germ, after an interval of several hours, varying according to the poison.

Here we have a clear distinction established between what may be called *bacterial infection* and *bacterial poisoning.* In the first form the living germ is implanted in the susceptible animal together with more or less of the *ptomaines* and other excretary matter. The germ goes on increasing at the expense of the animal tissues or fluids, and meanwhile throws out into the system increasing quantities of the chemical poisons, while by and by accumulate in quantity sufficient to produce a marked disturbance of the general health. In the bacterial poisoning on the other hand, whatever constitutional disorder is induced comes on at once, and if death fails to result, a steady improvement follows, for the simple *ptomaines* have no power of self-multiplication and increase, but are slowly eliminated from the body, and health is finally restored. With the *ptomaines* the effect will be exactly in ratio with the dose administered. The effects can therefore be graduated as accurately as those of any other purely chemical agent. Nor can these be the medium of the transferance of the disease to another victim. Thus with the sterilized *ptomaines* we may produce the symptoms and fatal result of an affection well known as a contagious one, yet without any power of propagating contagion or reproducing the disease in another subject.

*The importance of the ptomaines in disease.* It is not sought to deny the importance of the bacteria or other living germs in the causation of disease. It is conceded that they are fundamental, for without them the *ptomaines* cannot exist, while their presence in the system is directly productive of the ptomaines. Neither is it intended to depreciate the importance of accessary causes, atmospheric, electrical, telluric, climatic, hygienic, &c. All of these have their place in preparing the animal system for a defeat in the encounter with the disease-germ, and no less in acting on the germ itself in securing an increase or a decrease of its potency. It is more pertinent to our present purpose to draw especial attention to the action of the excretions of the living germ.

That the *ptomaines* are the most direct morbific agents may be deduced from various considerations:

1st. They are in many cases deadly poisons, though entirely separated from the living germs that produce them. They are therefore calculated to lay the tissues open to the destructive action of the germs themselves. Bacteria and fungi that produce no poisonous products are usually not injurious to the animal system. We find them in all exposed surfaces, in the mucous canals, and even in the superficial layers of the gastro-intestinal mucosa, yet no harm comes from their presence, they act mainly as scavengers, using up and removing dead matter which would tend to become superfluous or hurtful. In the tissues or blood of perfectly healthy animals, they are never found (Meissner).

Even when directly injected into the blood, some disease-germs perish promptly without producing either local or general disturbance. Such is the case with lung plague, and in rabbits and sheep, with rabies. In certain cases where they do attack the tissues, an unhealthy state of the tissues seems to be essential to the success of the attack. This is very noticeable in ring worm and holds no less true for a number of the bacteria. There is in short a veritable antagonism maintained between the living germ, and the living corpuscles and nuclei in the blood and tissues. When introduced in small quantity into the blood only, the contact of the few disease-germs in rapid succession with countless myriads of living blood globules places the germs at a disadvantage, and in place of destroying and assimilating the globules, they are themselves destroyed. When they do gain access to the blood and survive there, it is usually through the lymph channels in which the corpuscles are fewer and less elaborately organized, and where the flow is more tardy, so that their contest is with a relatively very small number of globules, until the bacteria have attained to great numerical force. Examples of this especial implication of the lymphatic system are seen in glanders, tuberculosis, strangles, swine-plague, anthrax, septic poisoning, &c.

In all such cases, as well as in the extension of the disease deeply from a morbid process on the surface, the role of a poisonous *ptomaine* is very potent. Exerted first on the nuclei of the tissues and the tissues themselves, it impairs or abolishes their natural functions and lays them open to chemical change. Under this lowered vitality they become an easy prey to the vigorous germ on which its own products have no such deleterious effect. Later the multiplying germs enter the lymphatic channels and similarly overcome the enfeebled lymph globules, and it is only when owing to the excessive increase of the germs that an excess of the morbid products is passed on into the blood, that the blood globules fail to hold their own, and the germ gains a status in the vital fluid.

2d. In keeping with this is the consideration that the disease-germ is a solid body and will not readily pass into the healthy solid tissues, so as to attack and impair their vitality. They may debilitate them by abstraction of oxygen and other elements, but no one can conceive of this having an equally enfeebling and destructive effect with the entrance in the untriunt fluid of the tissue of a soluble *ptomaine* endowed with the toxic power of a potent organic alkaloid. The soluble character of the *ptomaine* then is especially favorable to the establishing of its toxic action. Thus it becomes the forerunner of the germ and paves the way for its ultimate triumph.

3d. The acquired power of resisting a contagious disease, after a first attack has been endured, further sustains this doctrine of the action of the *ptomaines.* If this resistance were due to the absence of some necessary food of the bacterium, or to the presence of some substance poisonous to it, the germ would no more grow in the soup made from such tissues than in the living tissues themselves. The fact, that, it does grow in such soup, intimates that the resistance is vital and not chemical. It is difficult to understand the existence of a vital antagonism to a living organism which but assimilates its own material from the vital fluids and throws out its excreta. Much more conceivable is the acquired insusceptibility to the poisonous action of the *ptomaines.*

4th. The same doctrine is sustained by the protective action of a purely local contagious disease. As I pointed out in my report to the Department of Agriculture for 1880–1, the inoculation of lung plague in the tail, produces no active inflammation nor exudation save in the tail, yet the lungs, the normal seat of the disease, afterward resist the poison. Now if the bacteria actually reached the lungs, after the inoculation, what is to hinder the development of the local disease in this, the normal seat of its election. But taking into account that the same virus injected into the blood produces no local disease whatever, but is destroyed in the vital fluid, we are furnished, with substantial evidence that the living germ does not reach the lung at all after insertion in the tail, but perishes in the intervening blood vessels. What then reaches and protects the lungs? Manifestly the chemical products.

5th. A fifth argument in the same direction is deduced from the immunity that inheres to the fœtus, which was at an advanced stage of gestation, when its dam passed through an attack of a given contagious disease. The blood of the mother may swarm with bacteria, while that of the fœtus is entirely clear, yet the system of that fœtus is afterward as refractory to that poison as is the system of its dam. What then has entered the body of the fœtus so as to protect it? Manifestly the chemical products.

*Significance of the above in relation to Prophylaxis.*—It follows from the above that the chemical products may be used in self-limiting or non-recurring contagious diseases with the view of conferring immunity on the system. The debilitating poison (*ptomaines*), which paves the way for the triumph of the germ, is a simple chemical poison to which the system may become insusceptible just as the animal economy becomes by use unimpressible by alcohol, tobacco, opium, Indian hemp, arsenic, or other poison. The resistence inheres in the living tissues alone and is therefore a vital power. If therefore we can accustom the system to the chemical products of a given disease-germ, so that its nuclei will not be any longer depressed nor enfeebled by contact with such products, we place the system in a state of comparative immunity from the attacks of that germ.

As early as 1880 this was resorted to by Toussaint, who used first, filtered and then heated anthrax fluids with gratifying success, though his explanation, viz: a resulting condensation of the lymphatic glands—was an altogether untenable one. Struck with Toussaint's results, and realizing a different and more satisfactory explanation, I in the same year applied the method in swine plague, as set forth in Department of Agriculture Report for 1880–1. Since that time I have met with equal success in its application to lung plague and anthrax, but I must defer any detailed account of these to a future paper.

